# IngredSAM: Open-World Food Ingredient Segmentation via a Single Image Prompt

**DOI:** 10.3390/jimaging10120305

**Published:** 2024-11-26

**Authors:** Leyi Chen, Bowen Wang, Jiaxin Zhang

**Affiliations:** 1 College of Food Science and Nutritional Engineering, China Agricultural University, Beijing 100083, China; s20233061263@cau.edu.cn; 2 D3 Center, Osaka University, 2-1, Yamadaoka, Osaka 5650871, Japan; wang@ids.osaka-u.ac.jp; 3 Architecture and Design College, Nanchang University, No. 999, Xuefu Avenue, Honggutan New District, Nanchang 330031, China

**Keywords:** SAM, open-world food segmentation, visual prompting

## Abstract

Food semantic segmentation is of great significance in the field of computer vision and artificial intelligence, especially in the application of food image analysis. Due to the complexity and variety of food, it is difficult to effectively handle this task using supervised methods. Thus, we introduce IngredSAM, a novel approach for open-world food ingredient semantic segmentation, extending the capabilities of the Segment Anything Model (SAM). Utilizing visual foundation models (VFMs) and prompt engineering, IngredSAM leverages discriminative and matchable semantic features between a single clean image prompt of specific ingredients and open-world images to guide the generation of accurate segmentation masks in real-world scenarios. This method addresses the challenges of traditional supervised models in dealing with the diverse appearances and class imbalances of food ingredients. Our framework demonstrates significant advancements in the segmentation of food ingredients without any training process, achieving 2.85% and 6.01% better performance than previous state-of-the-art methods on both FoodSeg103 and UECFoodPix datasets. IngredSAM exemplifies a successful application of one-shot, open-world segmentation, paving the way for downstream applications such as enhancements in nutritional analysis and consumer dietary trend monitoring.

## 1. Introduction

Food semantic segmentation [1,2,3,4,5,6] enables the automated identification and differentiation of various food ingredients in images, which is valuable for nutritional analysis (e.g., 40% sugars, 30% fats, 30% proteins). This capability underpins the development of applications such as fitness diet recommendations, nutritional supplement suggestions, automated monitoring of dietary and nutritional intake [7], and the detection of consumer dietary trends [8], thus advancing intelligent monitoring and management within the food industry.

Traditional supervised deep learning methods [9,10,11,12] have demonstrated impressive segmentation performance on existing food datasets. However, these methods require large amounts of labeled data for training and are limited to recognizing only categories seen during training. The immense diversity in food types and appearances [13], combined with issues such as class imbalances [11], complex ingredient compositions, and amorphous or low-contrast food appearances, presents significant challenges for these models, hindering their effectiveness in accurately segmenting food images.

Recently, Meta AI introduced the Segment Anything Model (SAM) [14], trained on an extensive dataset comprising over 1 billion masks across 11 million licensed images. This model pioneers a groundbreaking approach to segmentation through a promptable framework, marking a new era in visual foundation models. This ambitious achievement represents a significant advance toward comprehensive perceptual recognition of objects in the real world. Notably, SAM is particularly effective for handling the challenges associated with diverse and visually distinct food ingredients.

SAM is designed to address open-world segmentation challenges, employing prompt engineering [15] from NLP to guide the model towards the desired outputs [16]. It uses points, bounding boxes, masks, and text prompts to control appropriate mask generation, effectively adapting to real-world tasks. However, since SAM performs open-world image segmentation, the generated food masks cannot differentiate between food ingredients, making them unsuitable for direct application. Additionally, the existing datasets’ annotations of food ingredients fail to describe all types of food in the world, which poses further challenges for food ingredient segmentation. To address this, we propose **IngredSAM**, a one-shot, promptable, open-world food ingredient segmentation approach, which reasonably extends SAM’s robust capabilities to the semantic segmentation of food ingredient categories while avoiding the category restrictions of existing food segmentation datasets.

**Figure 1:**
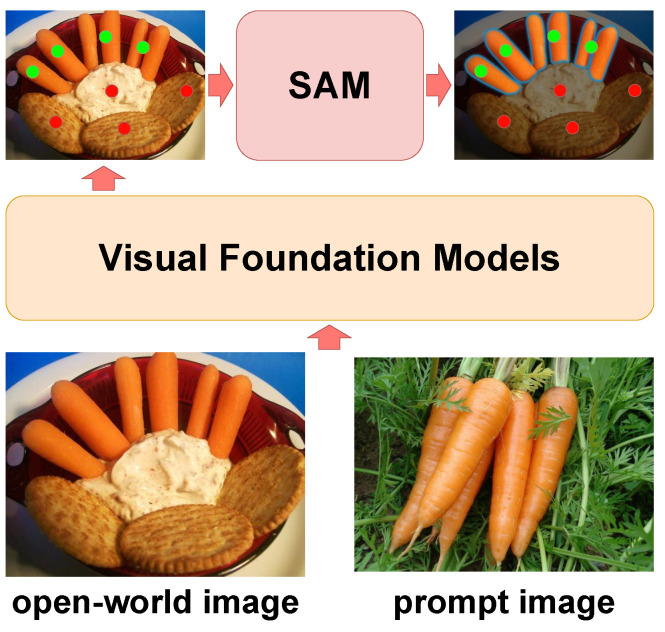
The prompt image provides the segmentation target, while the Visual Foundation Models generate point prompts for the open-world image.

Drawing inspiration from existing visual foundation models (VFMs) [17,18,19,20,21] and SAM, we introduce a flexible, distinguishable food category prompt image—essentially a clean image of specific ingredients. As shown in Figure 1, by leveraging multiple VFMs such as DINOv2 [19] and MAE [20], we obtain discriminative matchable semantic feature between the prompt image and open-world image features. We compute pixel-level similarities between the features of the prompt image and the open-world image to guide the generation of point prompts, which represent the spatial location of the food ingredients in the open-world image. Finally, this prompts SAM to generate a mask for the food ingredients in the open-world image.

In summary, we propose an open-world food ingredient segmentation approach based on the representational capabilities of visual foundation models and the powerful segmentation knowledge of SAM, enabling train-free segmentation of any food ingredient. The purpose of the VFMs is to guide the generation of appropriate point prompts to prompt SAM in creating the suitable masks. Through comprehensive evaluation of the FoodSeg103 [1] and UECFoodPix Complete [11] food segmentation benchmarks, IngredSAM outperforms the state-of-the-art methods on both datasets. As a potent method for open-world food ingredients semantic segmentation, IngredSAM provides an effective basis for downstream applications such as fitness diet recommendations, nutritional supplement suggestions, automated monitoring of dietary and nutritional intake, and detection of consumer dietary trends.

Our contributions are as follows:

- We introduce a new one-shot open-word food ingredient segmentation framework named IngredSAM, which utilizes the discriminative representational capabilities of VFMs and the powerful segmentation knowledge of SAM. With this framework, we can achieve train-free segmentation of any food ingredient.
- We introduce multiple visual encoder feature fusion to generate better prompt points for the continued SAM implementation.
- We validated IngredSAM on widely used food segmentation datasets, such as FoodSeg103 and UECFoodPix Complete. Compared to supervised expert models, SAM variants, and open-world approaches, IngredSAM demonstrated superior performance.

## 2. Related Work

### 2.1. Visual Foundation Models

The emergence of large language models with strong generalization capabilities [15,17,22,23] in NLP has inspired the development of large visual models in computer vision. Among these, CLIP [17] aligns image and text feature spaces through contrastive learning on vast numbers of image-text pairs, demonstrating powerful zero-shot generalization capabilities in various downstream visual tasks [24,25], such as open-world segmentation [26,27]. SAM trains a large segmentation model based on prompts on one billion masks, introducing prompts with sparse (points, boxes, text) and dense (mask) inputs. This flexible prompting mode can facilitate a wide range of downstream applications such as object tracking [24,28,29], image segmentation [30,31,32,33,34,35,36], and 3D reconstruction [37]. Additionally, VFMs like DINOv2 [19], CLIP, and MAE [20] learn strong object-level or dense semantic representations. These powerful representations effectively characterize different object attributes in complex scenes.

### 2.2. Food Segmentation

Food ingredient segmentation is an essential technology for realizing health applications such as recipes, nutrition [38], and calorie content [39]. Compared to general object semantic segmentation, food image segmentation poses greater challenges due to the enormous diversity of food appearances [13] and class imbalances [40]. For images containing multiple types of food, segmentation is a necessary prerequisite for dietary assessment systems. Segmenting overlapping, amorphous, or low-contrast foods that lack distinct color or texture features is particularly challenging. Additionally, lighting conditions that introduce shadows and reflections can adversely affect segmentation performance. Overall, the complex visual characteristics and arrangements of food make this field exceptionally demanding. Advanced segmentation techniques capable of handling food variability in unconstrained environments remain crucial for practical deployment.

Wu et al. [1] introduced ReLeM, which, by integrating it into semantic segmentation models, reduced the high intra-class variance from ingredients subjected to different cooking methods [2,10]. Wang et al. [3] combined the Swin Transformer and PPM module in their STPPN model to achieve state-of-the-art food segmentation performance. Honbu et al. [4] explored few-shot segmentation methods for undiscovered food categories in USFoodSeg. Sinha et al. [5] used Transformers and convolutional backbones to transfer visual knowledge into food segmentation.

To the best of our knowledge, prior research on open-world food ingredient segmentation is quite limited. Our work focuses on a straightforward approach to segmenting and distinguishing food ingredients in open-world food images without requiring any training.

## 3. Method

**Figure 2:**
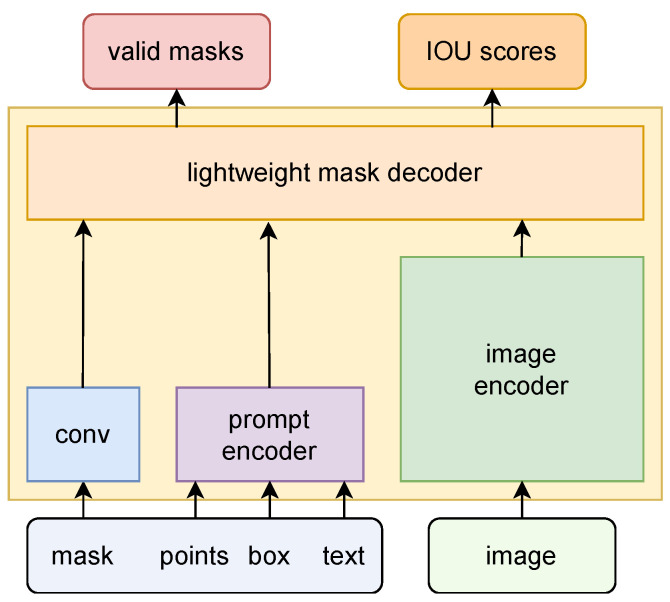
Pipeline of SAM. Mask, points, box, and text are four types of prompts.

**Figure 3:**
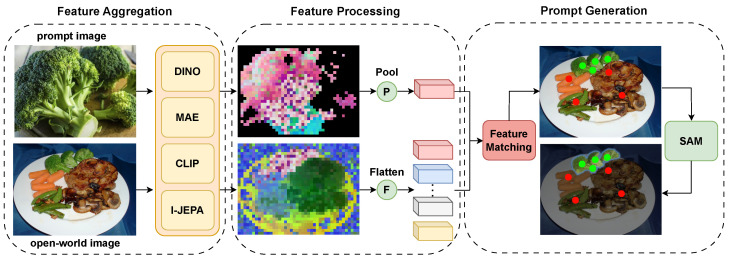
IngredSAM Architecture: Our model is divided into three stages: Feature Aggregation, Feature Processing, and Prompt Generation. The final stage outputs point prompts used to prompt SAM to generate reasonable masks for the open-world image.

As shown in Figure 2, we first detail the training and inference processes of SAM. Then, we introduce the proposed IngredSAM, as shown in Figure 3. Given a clear target food image prompt and an open-world image, IngredSAM can output point prompts representing the target food locations in the open-world image. SAM then uses the point prompts to segment the target food mask.

### 3.1. Preliminary

SAM represents the first application of VFM in the domain of image segmentation. As shown in Figure 2, the proposed model comprises four key components: an image encoder, a prompt encoder, a convolutional network, and a lightweight mask decoder module. The image encoder employs an MAE pre-trained Vision Transformer (ViT) [41] to efficiently extract fine-grained visual features from input images, available in three specific pre-trained image encoder configurations: ViT-B (91M parameters), ViT-L (308M parameters), and ViT-H (636M parameters). The convolutional network and prompt encoder support four types of prompt inputs: mask, points, box, and text. Dense prompt masks are embedded using convolutional layers and combined element-wise with image embeddings. Positional encodings [42] are added to the learned embeddings for points and boxes, specific to each prompt type. For free-form text prompts, embeddings are generated using a pre-trained text encoder from CLIP [17]. The mask decoder module, adopting a Transformer-based [43] architecture, applies self-attention to the prompts and cross-attention between the prompts and the output of the image encoder. The model ultimately outputs pixel-level mask probabilities and a predicted joint Intersection over the Union (IoU) metric. Crucially, the mask decoder can generate multiple mask outputs to accommodate the inherent ambiguity in the prompts, with the default configuration predicting three masks per prompt. Notably, the image encoder extracts features from each input image only once, allowing cached image embeddings to be reused across different prompts for the same image. Separating costly image inference from lightweight prompt interactions enables new interactive use cases such as real-time mobile augmented reality prompts. SAM was trained on a super large-scale dataset containing over 11 million images and 1 billion masks, demonstrating its robust and powerful zero-shot transfer capabilities.

### 3.2. IngredSAM

The process of our method is illustrated in Figure 3. The proposed IngredSAM framework consists of three parts: Feature Aggregation, Feature Processing, and Prompt Generation. The Feature Aggregation module is used to extract aggregated features from multiple visual encoders for both the prompt image and the open-world image. During the Feature Processing stage, the aggregated feature map of the prompt image, based on the foreground mask provided by TSDN [44], obtains its foreground-pooled average features, F-PI, while the open-world image undergoes a flattening operation to obtain flattened features, F-OI, for use in the subsequent feature matching process. In the Prompt Generation stage, F-PI and F-OI perform feature matching to calculate their similarity, which guides the generation of positive and negative point prompts for prompting SAM to perform image segmentation. These three components will be explained in detail in the subsequent sections.

#### 3.2.1. Feature Aggregation

We aim to obtain robust feature representations of the prompt image pi and open-world image oi to effectively capture the visual semantic information in both sets of images and ensure consistent semantic information can be found between them. Typically, image feature representations can be divided into multiple levels, for which we have designed a multi-encoder feature fusion process for different levels. We choose DINOv2 [19], known for its excellent discriminative features, MAE [20], known for its fine-grained features, CLIP [17], known for its strong semantic knowledge, and I-JEPA [21], known for its scene inference capabilities within the feature, as our multi-level feature encoders. After extracting robust, rich, and semantically consistent feature maps, the feature aggregation process can be formalized as follows:

(1) $$f_{pi}=Cat\left(f_{pi}^{D},f_{pi}^{M},f_{pi}^{C},f_{pi}^{I}\right)$$

(2) $$f_{oi}=Cat\left(f_{oi}^{D},f_{oi}^{M},f_{oi}^{C},f_{oi}^{I}\right)$$

where $f_{*}^{D}$, $f_{*}^{M}$, $f_{*}^{C}$, $f_{*}^{I}$ represent the feature maps output by DINOv2, MAE, CLIP, and I-JEPA, respectively, and $f_{pi}$ and $f_{oi}$, respectively, denote the aggregated feature maps of the prompt image and the open-world image. The features from these encoders are concatenated along the channel dimension.

#### 3.2.2. Feature Processing

We need to transform the aggregated feature maps $f_{pi}$ and $f_{oi}$ into pooled and flattened features, respectively, for subsequent feature matching. To obtain the discriminative object features $z_{pi}$ of the prompt image, we employ an unsupervised foreground segmentation method, TSDN, to obtain its foreground mask. TSDN leverages a Confidence-aware Saliency Distilling (CSD) strategy to progressively distill saliency knowledge from easy to hard samples, ensuring robust saliency detection even in challenging scenarios. Additionally, its Boundary-aware Texture Matching (BTM) strategy refines saliency boundaries by aligning texture patterns near the predicted boundaries, producing high-quality pseudo masks. These refined masks allow us to extract precise discriminative foreground object semantics using the average pooling operator (Avgpool):

(3) $$z_{pi}=Avgpool\left(f_{pi}⊙M\right)$$

where ⊙ denotes pixel-wise multiplication. The foreground object mask *M* is directly obtained by the unsupervised TSDN method.

The flattened aggregated feature map of the open-world image is represented as:

(4) $$z_{oi}=Flatten\left(f_{oi}\right)$$

#### 3.2.3. Prompt Generation

During the feature matching stage, by calculating the pixel-level similarity between $z_{pi}\in R^{1\times d}$ and $z_{oi}\in R^{HW\times d}$, where *H* and *W* represent the resolution of the spatial feature map of $f_{oi}$, we can obtain a semantic similarity distribution on the open-world image that corresponds to the food prompt represented by the prompt image, which then guides the generation of positive and negative point prompts. Specifically, first, we calculate the similarity score between $z_{pi}$ and $z_{oi}$ using cosine similarity. Secondly, we use the TopK algorithm to select points in the open-world image that are most semantically similar to the prompt image (food prompt):

(5) $$S=z_{pi}⊗z_{oi}^{T},\;P_{pos}=TopK\left(S\right)\in R^{K}$$

where ⊗ denotes matrix multiplication. As shown in Figure 3, the foreground food in the prompt image and the food to be segmented in the open-world image maintain good semantic consistency, ensuring the effectiveness of our TopK algorithm. Finally, we further refine $P_{pos}$ into *c* cluster centers as positive point prompts for SAM. Additionally, using the same pipeline, we also select *K* points of least similar to the $z_{pi}$ and cluster them into *c* cluster centers as negative point prompts for SAM. Following IPSeg [45], K=32 and c=4 are set. The generated positive and negative point prompts, along with the open-world image oi, are sent to SAM to predict the final food segmentation results.

Our pipeline can be summarized as follows. First, we use a visual encoder to extract image features from the prompt image and perform average pooling on the feature to obtain a feature vector representing the prompt image. Similarly, the visual encoder extracts image features from the open-world image, but without pooling, retaining the spatial resolution to get a feature map representing the open-world image. Next, we calculate the similarity between the feature vector of the prompt image and each pixel feature on the feature map of the open-world image. This similarity represents the degree of resemblance between the prompt image and specific regions of the open-world image. In real-time applications, the positive point prompts are derived by identifying regions with high similarity scores, typically corresponding to the target ingredient, while negative point prompts are generated from regions with low similarity scores, such as the background or other unrelated objects. These prompts are dynamically adjusted based on predefined thresholds or application-specific criteria to ensure robust guidance for SAM. Finally, the point prompts, combined with the open-world image, are input into SAM, which utilizes this information to generate the final ingredient mask that accurately identifies the ingredient depicted by the prompt image.

## 4. Experiment

### 4.1. Datasets

**UECFoodPix Complete** [11] was released by the University of Electro-Communications in 2020. It includes 102 types of dishes, comprising 9000 training images and 1000 test images. Segmentation masks were obtained semi-automatically using GrabCut, which segments images based on user-initialized seeds [46]. The automatically generated masks were further refined by human annotators according to a set of predefined rules [47].

**Figure 4:**
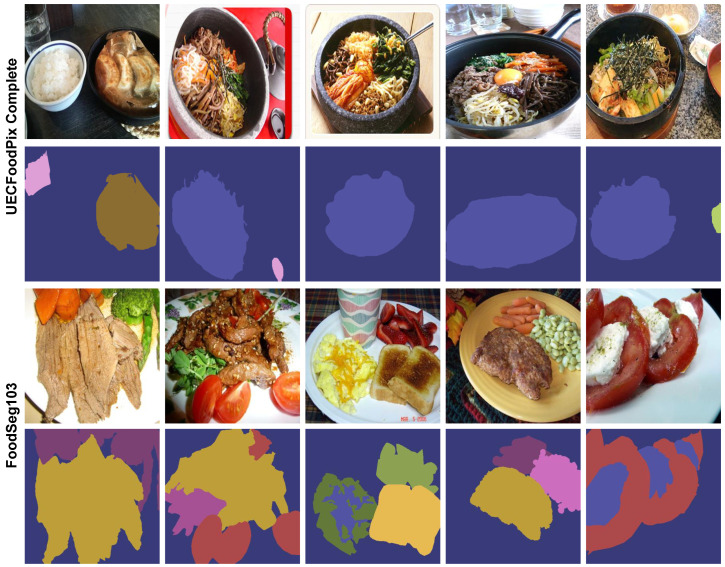
UECFoodPix Complete and FoodSeg103 datasets samples: it can be observed that the UECFoodPix Complete dataset does not provide detailed annotations for food ingredients, whereas FoodSeg103 includes detailed annotated masks for all ingredients.

**FoodSeg103** [1] is a dataset recently designed for food image segmentation, consisting of 7118 images annotated with 103 types of food ingredients. FoodSeg103 aims to annotate food ingredients more finely, capturing individual components of each food. Specifically, the training set contains 4983 images and 29,530 ingredient masks, while the test set comprises 2135 images and 12,567 ingredient masks. They were obtained through manual annotation. Compared to UECFoodPix Complete, FoodSeg103 has proven to be a more challenging benchmark for food image segmentation, and it aligns more closely with our objectives. Unlike UECFoodPix Complete, which annotates entire food, FoodSeg103 provides detailed annotations of individual food ingredients, as explained in Figure 4.

### 4.2. Implementation Details

We conducted experiments on the aforementioned datasets using an NVIDIA GeForce RTX A6000 GPU. For the UECFoodPix Complete dataset, we randomly selected an image from the same category as the prompt image as the open-world image. For the FoodSeg103 dataset, we created 103 clear food images of different food ingredients (one of which represents other ingredients) to serve as prompt images, with all images from FoodSeg103 acting as open-world images. We standardized the images to a resolution of 224×224 pixels for input into the IngredSAM. We selected DINOv2-ViT-L/14, MAE-ViT-H/14, CLIP-ViT-L/14, and I-JEPA-ViT-H/14 as the multi-level visual feature extractors. Regarding the unsupervised foreground detection method TSDN, we used the same hyperparameters as in the original paper to obtain the foreground mask for the prompt image. We employed ViT-H as SAM’s image encoder and used the same hyperparameters as the original paper. The entire pipeline involves no training.

**Evaluation Metrics**: We employ common metrics to assess model performance, such as mean Intersection over Union ($IoU_{mean}$) for each image. mIoU is a standard metric in semantic segmentation that evaluates the overlap and conjunction between inference and ground truth, as described below:

(6) $$IoU_{mean}=\frac{1}{N}\sum_{i=1}^{N}\frac{TP_{i}}{TP_{i}+FP_{i}+FN_{i}}$$

where *N* is the number of classes and $TP_{i}$, $FP_{i}$, and $FN_{i}$ are described as follows:

- True Positive ($TP_{i}$) represents the number of pixels that are correctly classified as class *i*.
- False Positive ($FP_{i}$) denotes the number of pixels that are wrongly classified as class *i*.
- False Negative ($FN_{i}$) is the number of pixels that are wrongly classified as other classes while their true labels are class *i*.

We also introduced the calculation of the $IoU_{i}$ for specific food ingredient *i*:

(7) $$IoU_{i}=\frac{TP_{i}}{TP_{i}+FP_{i}+FN_{i}}$$

### 4.3. Main Results

We compared IngredSAM with state-of-the-art supervised models and open-world segmentation methods. Supervised methods like FPN [9], CCNet [2], and SETR [10] leverage advanced backbone architectures or transformer designs for accurate segmentation, while methods such as Bayesian DeeplabV3+ [48] and GourmetNet [49] incorporate domain-specific adaptations to enhance performance. Open-world methods like SegGPT [50] and IPSeg [45] focus on semantic representation matching but struggle with the fine-grained diversity of food ingredients. IngredSAM outperformed all methods on FoodSeg103 and UECFoodPix Complete datasets, demonstrating its ability to address complex ingredient segmentation challenges with a robust, train-free framework.

#### 4.3.1. Comparison with Supervised Models

In this section, we evaluate the performance of our proposed IngredSAM model in comparison to various state-of-the-art supervised methods across two significant datasets: FoodSeg103 and UECFoodPix Complete.

Evaluation on FoodSeg103 Dataset: As demonstrated in Table 1, IngredSAM sets a new benchmark by achieving the highest mean Intersection over Union (mIoU) of 48.78%, surpassing the previous best supervised SETR model which recorded a 45.10% mIoU. Notably, IngredSAM shows substantial improvements across the top five ingredients with a particularly strong performance in the “bread” and “tomato” categories, reflecting an mIoU of 69.17% and 58.02%, respectively. These results substantiate IngredSAM’s superior segmentation accuracy and robustness in handling diverse food ingredients.

Evaluation of UECFoodPix Complete Dataset: Since the dataset does not distinguish detailed food components, the segmentation task is simpler. As indicated in Table 2, IngredSAM continues to outperform existing models with a remarkable mIoU of 70.21% on the UECFoodPix Complete dataset. It shows unprecedented performance, especially in the “rice” category, achieving an mIoU of 76.47%. IngredSAM’s enhancement in performance metrics across various food items confirms its effectiveness in segmenting complex scenes with high accuracy and consistency.

#### 4.3.2. Comparison with Open-World Methods

In further evaluating our IngredSAM model, we also compare its performance with SAM variants that leverage image prompt for semantic segmentation tasks.

Performance Comparison: The comparative analysis presented in Table 1 illustrates the superior capabilities of IngredSAM against SAM variants PerSAM and IPSeg. IngredSAM demonstrates a notable increase in the mean Intersection over Union (mIoU) across all food categories on the FoodSeg103 dataset, achieving a 103-mean of 48.78%. This is significantly higher than the IPSeg, which previously held the best performance among other methods with a 103-mean of 45.93%. PerSAM, another SAM series using image prompts, shows substantially lower performance with a 103-mean of 31.81%. We also compare our method with some open-world segmentation methods, e.g., Painter, SegGPT, and DeLVM, which show worse performance in ingredient segmentation. Note that all methods use an image prompt to introduce category information for performing open-world segmentation. Overall, IngredSAM’s performance is more efficient, demonstrating its potential for broader application in real-world scenarios where not all food annotations are available.

### 4.4. Qualitative Results

**Figure 5:**
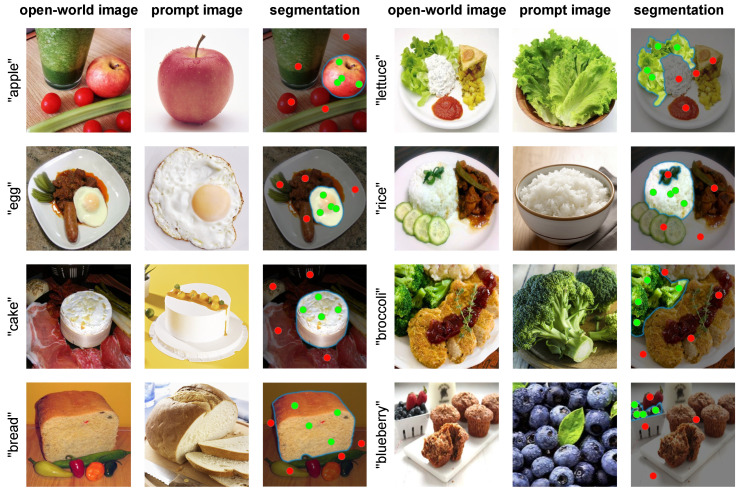
IngredSAM Segmentation Visualization Results: It can be seen that the food ingredients represented by the prompt image are completely segmented in the open-world image.

In Figure 5, we showcase the visualization results of the IngredSAM network. These visualizations highlight the network’s capability to effectively segment food ingredients in various complex scenes. Visually, this demonstrates the effectiveness of our method. Particularly noteworthy is the network’s performance in complex scenes involving objects in very small areas, such as those labeled “blueberry”. Our IngredSAM network accurately segmented the target objects hidden in a bowl filled with strawberries and blueberries, underscoring its proficiency in correctly identifying objects in the open-world image that have semantic correspondences with those in the prompt image. This capability demonstrates the robustness and adaptability of the IngredSAM network in handling various challenging segmentation tasks.

### 4.5. Ablation Studies

In the ablation studies, we primarily explored the combined efficacy of multi-level visual encoders and the utility of background filtering algorithms.

#### 4.5.1. Combination of Visual Foundation Models

This section of our ablation study evaluates the impact of combining multiple VFMs to understand their collaborative effects on the performance of semantic segmentation in the FoodSeg103 dataset. The results in Table 3 clearly demonstrate that the integration of diverse VFMs leads to significant improvements in model performance. When only using DINOv2, it achieves an mIoU of 46.31%, showcasing its robust feature extraction capabilities and the best semantic discrimination and consistency. On the other hand, I-JEPA, designed for semantic discovery and representational reasoning rather than semantic discrimination and consistency, shows an mIoU of 43.27%, the lowest among the individual models, suggesting it might require combination with other models to fully leverage its capabilities. The combination of DINOv2 and any other model consistently results in better performance. Specifically, the combination of DINOv2 and MAE shows the best improvement, achieving an mIoU of 47.96%. This suggests that the complementary nature of DINOv2’s strong semantic features and MAE’s fine-grained dense semantic features boost segmentation accuracy. The integration of all four VFMs—DINOv2, MAE, CLIP, and I-JEPA—yields the highest mIoU of 48.78%. This combination harnesses the distinct and powerful capabilities of each model, from DINOv2’s discriminative and consistent representation, MAE’s fine-grained representation, and CLIP’s extensive semantic knowledge, to I-JEPA’s semantic reasoning abilities, providing a comprehensive, effective semantic representation. This result underlines the importance of leveraging multiple levels of visual understanding, confirming that the synergy between different VFMs can significantly enhance performance.

#### 4.5.2. Impact of Background Noise

**Figure 6:**
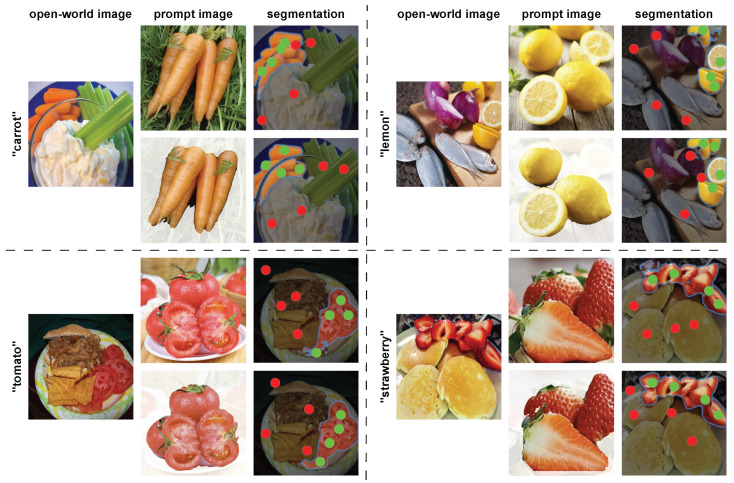
Visualization of the effectiveness of using a background filtering algorithm.

In Figure 6, we can see that the background filtering algorithm effectively filters out the background, resulting in clean and clear food in the prompt image. Overall, all prompt images that utilized the background filtering algorithm produced better point prompts that led to better segmentation results and avoided confusion. In the segmentation of carrots, we can observe that the positive point prompts generated by the prompt image without the background filtering algorithm, although concentrated around the carrot area, are overly clustered at the edges of a transparent bowl. This mistakenly segments the bowl’s edge as part of the carrot ingredient. Conversely, the positive point prompts generated by the image prompt using the filtering algorithm is well-distributed around the carrot area, successfully avoiding incorrect segmentation of the bowl’s edges. In the segmentation of lemons and strawberries, the image prompt that used the filtering algorithm also generated positive point prompts on the food ingredients hidden behind in the photo, prompting SAM to produce excellent masks. In summary, all the visualization results demonstrate that the background filtering algorithm is necessary.

#### 4.5.3. Impact of Using Different Image Prompts

Using image prompts is a crucial component of our method, making it essential to evaluate the impact of different image prompt settings. As presented in Table 4, we conducted experiments to quantify this impact on the FoodSeg103 dataset. We tested three distinct prompt settings, each involving 103 clear food ingredient images derived from various raw images in the training set. Notably, Setting 1 is used for the main experiment. The results indicate minimal performance differences across the settings, suggesting that the choice of image prompts does not significantly affect performance and highlighting the robustness of our method.

## 5. Discussion

IngredSAM is an innovative one-shot, open-world segmentation framework that marks a significant advancement in the domain of food ingredient segmentation. In this section, we discuss the broader impact of this research and some limitations.

### 5.1. Potential Applications and Societal Value

IngredSAM has broad applications across various sectors. In the health industry, it can enhance nutritional tracking and diet management by providing precise segmentation of food ingredients from images. Consequentially, it can aid in the accurate calculation of nutritional intake. This could be applied to health apps to offer real-time dietary suggestions based on personal health data and daily eating preferences. In the industry aspect, IngredSAM could be utilized to automate ingredient detection in cooking shows or recipes, making it easier for viewers to understand the dishes.

The societal implications of IngredSAM are profound. By enabling more accurate and personalized nutritional tracking, it contributes to better public health outcomes. This technology can also play a crucial role in addressing global health issues such as obesity and malnutrition by providing insights into eating habits and food composition. Additionally, its nutritional analysis capabilities can also contribute to more efficient food production systems.

### 5.2. Comparison with Related Methods

Recent methods such as IPSeg, Painter, and PerSAM address open-world segmentation but are less effective for the unique challenges of food ingredient segmentation. IPSeg, while training-free, relies on single-image prompts and lacks the fine-grained semantic differentiation achieved by IngredSAM’s multi-model feature fusion using multi-encoders. Painter adopts a generalist inpainting framework, excelling in broad applications but struggling with tasks like food ingredient segmentation that require specialized optimization (Painter). PerSAM, focused on personalized segmentation, is limited to single-object scenarios and lacks the adaptability needed for complex ingredient compositions (PerSAM). In contrast, IngredSAM combines robust visual feature extraction with SAM’s promptable framework, enabling train-free, zero-shot segmentation of diverse food ingredients. This tailored design ensures superior segmentation quality and efficiency, as validated on FoodSeg103 and UECFoodPix Complete, where IngredSAM consistently outperforms these methods.

### 5.3. Inference Speed and Potential Model Acceleration Methods

Inference speed is vital for practical segmentation applications, particularly in resource-constrained environments. While IngredSAM achieves competitive FLOPs and accuracy compared to state-of-the-art models, future research can explore advanced model acceleration techniques to further enhance its efficiency. For instance, knowledge distillation approaches, such as CLIP-KD [59], could be applied to compress visual foundation models, reducing computational requirements while retaining robust feature extraction capabilities. Similarly, cross-image relational knowledge distillation techniques [60], proven effective in semantic segmentation tasks, may help optimize the segmentation components of IngredSAM, ensuring faster inference without compromising accuracy. These strategies provide promising avenues for improving IngredSAM’s applicability in real-time and large-scale scenarios.

### 5.4. Limitations and Future Work

While IngredSAM presents numerous advantages, several limitations warrant further exploration. The framework’s reliance on high-quality, discriminative images for inference and operation poses challenges in environments without discriminative images. Additionally, the ethical considerations regarding data privacy and the potential biases in dietary data collection need to be addressed to prevent skewed nutritional recommendations.

Additionally, the framework’s reliance on high-quality and discriminative image prompts for effective segmentation highlights an area for improvement. As discussed in Section 4.5.3, the choice of image prompts significantly impacts performance. However, the current approach lacks a mechanism for automated selection of optimal prompts tailored to specific segmentation scenarios. This limitation can hinder its robustness in diverse or uncontrolled environments, where ideal prompts may not be available.

Enhancing the model’s capability to accurately segment visually similar or overlapping ingredients is essential. Advanced strategies, such as integrating contextual information or leveraging multi-modal data (e.g., text descriptions), could help overcome these challenges. Second, developing methods for the automated selection of optimal image prompts can improve the framework’s robustness and adaptability to diverse scenarios.

Future work could focus on enhancing the robustness of IngredSAM against these challenges, such as improving performance on visually complex or low-contrast ingredients through advanced feature fusion techniques or adaptive prompt refinement. Furthermore, integrating IngredSAM with other state-of-the-art computer vision models, such as object detection frameworks or generative models for synthetic data augmentation, could expand its versatility in diverse scenarios. Real-time applications, including mobile-based food analysis tools or smart kitchen devices, represent another promising direction, enabling instant ingredient recognition and dietary feedback. Additionally, exploring partnerships with culinary experts and nutritionists could provide practical insights to refine the model’s applicability and effectiveness in real-world settings, ensuring its alignment with consumer needs and professional standards.

## 6. Conclusions

In this paper, we propose IngredSAM, which is marked as a pivotal step forward in the field of food ingredient semantic segmentation, highlighting the potential of one-shot, open-world approaches. The integration of visual foundation models with the innovative use of prompt-based segmentation has enabled our framework to perform robustly across standard food segmentation benchmarks, outperforming current supervised and other unsupervised methods. While IngredSAM successfully navigates the complexities of diverse food appearances and the limitations of existing datasets, future work will explore refining the precision of feature matching and extending the model’s utility to more granular nutritional analysis tasks. We anticipate that IngredSAM will inspire further research into flexible, adaptable models for complex segmentation challenges in various domains.

## Figures and Tables

**Table 1:** Compared to supervised (upper part) and open-world segmentation (lower part) methods on the FoodSeg103 dataset. Here, “bread”, “carrot”, “chicken”, “sauce”, and “tomato” represent the top five food ingredients with respect to the IoU. “103-mean” indicates the average IoU across all 103 food ingredients.

Method	Bread	Carrot	Chicken	Sauce	Tomato	103-Mean
FPN [9]	32.89	35.88	32.17	30.69	32.63	27.28
CCNet [2]	35.23	36.47	34.21	32.13	34.79	28.60
ReLeM-CCNet [1]	38.22	38.41	37.94	35.80	37.61	29.20
ReLeM-FPN-Finetune [1]	38.61	38.20	38.03	35.96	37.66	30.80
Window Attention [51]	38.76	38.62	36.49	36.51	38.70	31.40
Upernet [52]	49.71	51.27	46.22	44.16	47.67	39.80
STPPN [3]	51.23	52.17	48.09	44.13	48.37	40.30
CCNet-Finetune [53]	53.67	53.24	50.11	45.85	49.01	41.30
SETR [10]	63.41	62.17	54.31	51.91	53.92	45.10
PerSAM [54]	36.80	36.18	32.24	32.09	35.33	31.81
IPSeg [45]	67.31	65.71	54.23	55.17	60.16	45.93
Painter [55]	62.69	60.52	48.73	52.38	53.85	40.17
SegGPT [50]	44.53	51.49	32.68	50.06	41.64	36.46
DeLVM [56]	17.39	23.66	31.45	37.08	21.30	24.71
IngredSAM	**69.17**	**67.23**	**56.09**	**54.61**	**58.02**	**48.78**

**Table 2:** Performance of all comparison methods originates from paper [53]. Compared to supervised methods on the UECFoodPix Complete dataset. Here, “salad”, “beverage”, “soup”, “noodle”, and “rice” represent the top five food ingredients with respect to IoU. “102-mean” indicates the average IoU across all 102 food ingredients.

Method	Salad	Beverage	Soup	Noodle	Rice	102-Mean
deeplabV3+ [11,57]	57.62	58.32	56.31	56.71	58.91	55.50
YOLACT [58]	57.41	58.01	55.39	56.85	58.51	54.85
GourmetNet [49]	63.96	64.72	64.11	63.66	65.90	62.88
BayesianDeeplabv3+ [48]	65.77	67.13	55.98	65.14	68.43	64.20
IngredSAM	**73.35**	**75.26**	**72.44**	**72.73**	**76.47**	**70.21**

**Table 3:** Ablation studies on combinations of different VFMs.

#	Combination of VFMs				FoodSeg103
	DINOv2	MAE	CLIP	I-JEPA	mIoU
1	✓				46.31
2		✓			45.53
3			✓		44.82
4				✓	43.27
5	✓	✓			47.96
6	✓		✓		46.77
7	✓			✓	46.60
8	✓	✓	✓	✓	48.78

**Table 4:** Experiment for three different prompt settings on FoodSeg103 dataset. For each setting, we created 103 clear food ingredient images sourced from different raw images in the training set. Note that Setting-1 is used for the main experiment.

Prompt Setting	Bread	Carrot	Chicken	Sauce	Tomato	103-Mean
Setting 1	69.17	67.23	56.09	54.61	58.02	48.78
Setting 2	65.47	68.11	55.45	57.24	56.87	48.26
Setting 3	66.63	65.19	57.40	50.97	57.32	47.90

## Data Availability

Data and source code are available by contacting the corresponding author.
